# Serum Albumin Binding and Esterase Activity: Mechanistic Interactions with Organophosphates

**DOI:** 10.3390/molecules22071201

**Published:** 2017-07-18

**Authors:** Nikolay V. Goncharov, Daria A. Belinskaia, Vladimir I. Shmurak, Maxim A. Terpilowski, Richard O. Jenkins, Pavel V. Avdonin

**Affiliations:** 1 Research Institute of Hygiene, Occupational Pathology and Human Ecology, bld.93 p.o. Kuz’molovsky, Leningrad Region 188663, Russia; vladimir.shmurak@gmail.com; 2 Sechenov Institute of Evolutionary Physiology and Biochemistry, Russian Academy of Sciences, pr. Torez 44, St. Petersburg 194223, Russia; d_belinskaya@mail.ru (D.A.B.); maximtrp@gmail.com (M.A.T.); 3 School of Allied Health Sciences, De Montfort University, The Gateway, Leicester LE1 9BH, UK; roj@dmu.ac.uk; 4 Koltsov Institute of Developmental Biology, Russian Academy of Sciences, 26 Vavilova str., Moscow 119334, Russia; pvavdonin@ya.ru

**Keywords:** albumin, esterases, organophosphates, docking, molecular modeling

## Abstract

The albumin molecule, in contrast to many other plasma proteins, is not covered with a carbohydrate moiety and can bind and transport various molecules of endogenous and exogenous origin. The enzymatic activity of albumin, the existence of which many scientists perceive skeptically, is much less studied. In toxicology, understanding the mechanistic interactions of organophosphates with albumin is a special problem, and its solution could help in the development of new types of antidotes. In the present work, the history of the issue is briefly examined, then our in silico data on the interaction of human serum albumin with soman, as well as comparative in silico data of human and bovine serum albumin activities in relation to paraoxon, are presented. Information is given on the substrate specificity of albumin and we consider the possibility of its affiliation to certain classes in the nomenclature of enzymes.

## 1. Introduction

The first publications devoted to the study of serum albumin date back to the end of the 19th century [1,2]. By the middle of the 20th century, dozens of works were published every year, which became hundreds in the 1960s, and in the 1970s their number exceeded a thousand. Surprisingly, the high resolution three-dimensional structure of human serum albumin (HSA) was disclosed rather late, not until the 1990s [3,4]. A similar structure was obtained for bovine serum albumin (BSA) in 2012 [5]. Albumin is the main protein of mammalian blood, where its concentration range is 500–700 μM. It is synthesized in the liver at a rate of about 0.7 mg per hour (i.e., 10–15 mg per day); the half-life of HSA is 19–20 days [6]. Serum albumin is formed by one polypeptide chain, consisting of 585 and 583 amino acids (a.a.) for human and bovine albumin, respectively [5]. The structure of albumin is conservative in all mammals: the molecule consists of three homologous domains, each consisting of ten helices and can be divided into two subdomains (A and B) containing six and four helices, respectively, with the two subdomains connected by a long loop [7]. When an albumin molecule interacts with different substances, its three-dimensional structure is sufficiently flexible enough to cause effects, such as cooperativity and allosteric modulation, usually inherent in multimeric proteins [8,9]. The human and bovine albumin molecule has 17 disulfide bonds and one cysteine residue with a free SH group [7,10]. In the BSA molecule, there are frequent replacements of lysine residues with arginine residues at the Sudlow site I (Lys195 → Arg195, Lys199 → Arg199), which can affect its conformation since arginine side chains are more branched.

The albumin molecule is not covered with a carbohydrate moiety and can bind a wide variety of molecules and atoms: water and metal cations (Ca^2+^, Zn^2+^, Cu^2+^, Ni^2+^, Cd^2+^, Co^2+^, Pt^2+^, Au^+^ [11]), free fatty acids and fat soluble hormones, unconjugated bilirubin, bile salts, transferrin, nitric oxide, aspirin, warfarin, phenylbutazone, clofibrate, etc. [12]. By binding drugs and toxic substances, albumin largely determines their pharmaco- and toxicokinetics, through transport to target tissues or sites of their biotransformation. Binding occurs at two primary sites and several secondary sites, the number of which depends on the physico-chemical properties of the substances and the state of the albumin molecule. For fatty acids—the main ligand of albumin—there are seven binding sites, with the bound fatty acids changing the polarity and volume of drug binding sites [10].

Albumin is not only a passive but also an active participant in numerous pharmaco- or toxicokinetic processes. Firstly, we are interested in the interaction of albumin with esters. In numerous experiments, esterase or pseudo-esterase activity of albumin was demonstrated with respect to: α- and β-naphthyl acetate and *p*-nitrophenylacetate (NPA) [13,14,15], fatty acid esters [16], aspirin [17], ketoprofen glucuronide [18], cyclophosphamide [19], esters of nicotinic acid [20], octanoylghrelin [21], nitroacetanilide [22], nitrotrifluoroacetanilide [23], and organophosphorus compounds [24].

Acetylation is a characteristic example of the pseudo-esterase activity of albumin (pseudo-first order reaction), when the decrease in the ester substrate is due not to its hydrolysis, but to the formation of covalent bonds along many amino acid residues (sites) of the albumin molecule. It was found that acetylation of albumin by NPA can proceed along 82 a.a. (sites), of which 59 are lysines, 10serines, eight threonines, four tyrosines, and one asparagine [25]. NPA exhibits the greatest affinity for Tyr411, though adducts acetylated for lysine residues are more stable.

In toxicology, the problem of (pseudo) esterase activity of albumin with respect to organophosphates (OPs)—esters of phosphoric or phosphonic acids—is of great importance. The sites for the formation of covalent adducts of OPs with albumin—Tyr411 and Tyr150 [26,27,28]—have been established. In addition to (pseudo) esterase activity, albumin exhibits peroxidase activity against hydroperoxides of lipids [29,30,31] and many other activities. Despite the obvious successes of modern analytical methods and the desire of some scientists to prove with the help of these methods the absence of true esterase and other types of enzymatic activity in albumin, the results obtained do not unequivocally solve the problem. Until now, the mechanisms of interaction of different esters and other compounds with albumin remain undisclosed.

The aim of the work reported here was to analyze selected data obtained over decades, which, along with our own data, would allow us to substantiate not only the “pseudo” but also the true enzymatic activity of albumin, and to clarify the role of albumin in the toxicology of OPs. Information was collected not only on the esterase, but also on other enzymatic activities of albumin, in order to consider its possible place in the nomenclature of enzymes.

## 2. Methods

### 2.1. Preparation of 3D-models of Ligands and Proteins

Three-dimensional structures of four isomers of soman and other ligands were constructed and then minimized by the method of steepest descent [32] using the program HyperChem 8.0 [33]. For molecular docking of soman into the binding sites of albumin, the three-dimensional structure of HSA was obtained from the protein data bank (PDB) [34]; the structure code is 2bxd [10]. Molecules of water and ligands were removed from the structure, then the molecule of albumin was minimized by the method of steepest descent [32] using the software package Gromacs [35]. Two protonation states of Tyr150 and Tyr411 were used to study soman binding into these sites: protonated state Tyr-OH and deprotonated state Tyr-O^−^.

For molecular docking of paraoxon into the binding sites of HSA and BSA, three models of HSA (models 1–3) and three models of BSA (models 4–6) from the Protein Data Bank were used [34]. The data from the X-ray analysis of a HSA molecule with a warfarin molecule bound in the Tyr150 site (the code of the structure is 2bxd [10]) was used as Model-1. The data from X-ray analysis of a HSA molecule with an ibuprofen molecule bound in the Tyr411 site (the code of the structure is 2bxg [10]) was used as Model-2. Model-3 (“relaxed” HSA molecule) was obtained from Model-1 by steepest descent energy minimization [32] and further relaxation by molecular dynamics (MD). The data from X-ray analysis of a free BSA molecule (the code of the structure is 4f5s [7]) was used as Model-4. The data from X-ray analysis of a BSA molecule with a diethylene glycol molecule bound in the Tyr149 site and with a naproxen molecule bound in the Tyr410 site (structure code 4or0 [36]) was used as Model-5. Finally, the data from X-ray analysis of a BSA molecule with two molecules of 3,5-diiodosalicylic acid bound in the Tyr149 and Tyr410 sites (structure code 4jk4 [37]) was used as Model-6. Again, molecules of water and ligands were removed from the structures before starting the docking procedures.

### 2.2. Molecular Docking Procedure and Dissociation Constant Estimation

Molecular docking of ligands to albumin binding sites was performed using the Autodock4.0 software package [38]. The approach described by Li et al. [26] was used. In the studied binding site, an approximation grid of size 60 nodes in x-, y-, and z-directions and with a step of 0.0375 nm was constructed. Estimated energy of binding (deltaG) is used by Autodock software as the scoring function to select the optimal conformations, with dissociation constant (*K*_d_) calculated using this value and the formula *K*_d_ = exp(deltaG/(R*T)) [38]. The Lamarckian genetic algorithm for searching the optimal conformations was used [38]. The search procedure was repeated 50 times for each protein-ligand pair which resulted 50 optimal conformations. The final conformations of the complexes with RMSD less than 0.05 nm was combined into clusters and sorted by ascending *K*_d_ values. Thus, the result of molecular docking procedure was a set of the 3–6 most likely conformations of an albumin-ligand complex. Launches, 4–18, of conformational search converged into each of these conformations. The productivity of the obtained conformations (the possibility of covalent bond formation between a catalytic amino acid and a ligand (soman, *p*-nitrophenyl acetate or paraoxon) was estimated from the distance between the hydroxyl oxygen atom of a catalytic acid (or Nζ-atom in the case of lysines) and the phosphorus (or carboxyl carbon in the case of NPA) atom of a ligand molecule (dist(P(C)-O(N))). The conformations inside a cluster have the same value of dist(P(C)-O(N)) due to their small RMSD. To further estimate *K*_d_ and MD calculations, conformations corresponding to the productivity criterion were selected. If the productive complexes were not found by molecular docking, the cluster with the lowest energy value was chosen. The final value of *K*_d_ was calculated as the mean of all values of the constants obtained in this cluster, while the error was estimated as half the difference between the maximum and minimum values inside the cluster.

### 2.3. Molecular Dynamic Simulation

Conformational changes of ligand-enzyme complexes were calculated by the MD method using the GROMACS software package [35]. The complexes obtained by molecular docking were placed virtually into a periodic box filled with water molecules. To check the stability of soman-albumin complexes, short 1000 ps simulations were performed. To study the conformational changes of the complexes paraoxon-HSA(BSA), 10 ns MD simulation was applied. The time step was set to 2 fs. The single-point charge (SPC) water model was used to describe the water in the simulation [39]. Simulations were performed at a temperature of 300 K and a pressure of 1 bar. Temperature and pressure were kept constant using Berendsen temperature and pressure couplers, with coupling constants of 0.1 ps and 1.0 ps [40,41]. Before running the MD simulations, all the structures were minimized by steepest descent energy minimization [32] and equilibrated with 10 ps MD simulation.

## 3. Results

### 3.1. Esterase and Pseudoesterase Activity of Albumin: Theoretical Considerations on Kinetic Schemes and Constants

**Scheme 1:**
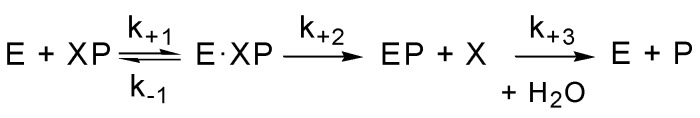
The reaction between esterase (E) and phosphorus ester XP [44].

**Scheme 2:**
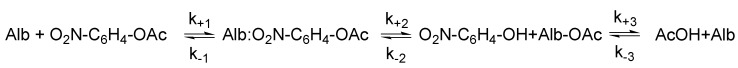
The reaction between human serum albumin (Alb) and NPA [45].

**Scheme 3:**
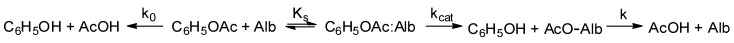
Spontaneous and albumin catalyzed hydrolysis of phenylacetate [46]. Here *k_0_* is the spontaneous hydrolysis constant, *K_s_* is the substrate constant (dissociation constant of the enzyme-substrate complex), *k_cat_* is the catalytic constant, *k* is the deacylation constant (dissociation constant of the acyl-albumin complex).

**Scheme 4:**
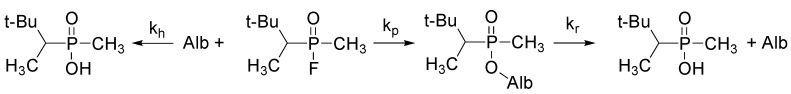
Spontaneous and albumin catalyzed hydrolysis of soman (3,3-dimethylbutan-2-ylmethylphosphonofluoride, C_7_H_16_FO_2_P) [26]. Here: *k_h_* is the constant of spontaneous hydrolysis, *k_p_* is the constant of phosphonylation of soman, *k_r_*—constant of albumin reactivation.

**Scheme 5:**
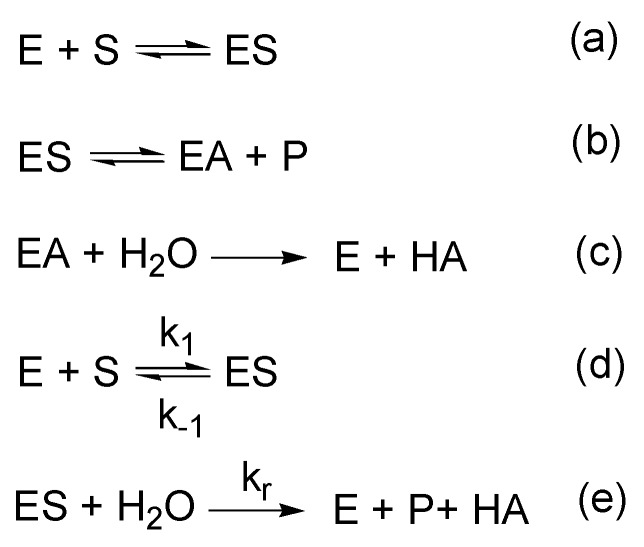
Scheme (a–c) illustrating all the main steps of interaction between acetylcholinesterase (E) and acetylcholine (S), and shortened scheme (d–e) [47], where E is an enzyme (esterase), S is the substrate, P is the product (choline), A is the acetyl moiety; *k_r_* is the constant of enzyme reactivation.

Enzymes increase reaction rates by lowering the energy of the transition state in two possible ways [42]: (1) reducing the free energy of the transition state by binding and orientation of the substrate; (2) altering the pathways of the reaction by producing multiple intermediate products. We proceed from the fact that albumin is capable of both ways of reducing the activation energy of the reaction. However, even if albumin carried out hydrolysis of esters only in the second way (changing the reaction pathway), it would still be reasonable to consider it an enzyme. The mechanism of a three-stage enzymatic reaction with the formation of a covalent adduct as an intermediate reaction product, has been described for so long that it has already become firmly established in classical textbooks on enzymology (see, for example, [43]). The *k*_2_ constant (equaling *k_cat_* in the case of *k*_2_ << *k*_3_) becomes a constant for the formation of a covalent adduct (e.g., an acylenzyme), an additional kinetic constant *k*_3_ (equaling *k_cat_* in the case of *k*_2_ >> *k*_3_) is introduced, which usually characterizes the limiting stage of the enzymatic reaction. In the case of acylation *k*_3_ describes the rate of hydrolysis itself, i.e., transition of an acyl group from a protein molecule to a water molecule (Scheme 1, Scheme 2, Scheme 3, Scheme 4 and Scheme 5) [26,44,45,46,47]. The design of the schemes and the symbols for the kinetic constants are similar, but the meaning can be rather different. The rates of acylation and deacylation for different substrates vary greatly, and it is principally important for discrimination between true- and pseudoesterase activities to introduce into the scheme the rate constant *k_+_*_2_ and then to calculate it; also, it is important to realize that this stage is reversible.

Taking this into account, Scheme 2 of Ascenzi and co-authors [45] is full-fledged and the only one which meets the eligibility criteria for discrimination of esterases. There has been much debate about the edge from which one can speak of “true” catalysis (esterase activity), or of pseudocatalysis (pseudo-esterase activity). The borders are still not established, and there is no regularity in the nomenclature of hydrolases and other groups of enzymes. Substrate specificity is an important factor for assigning enzymes to a particular group (class, subclass, sub-subclass), and problems often arise when there are two or more binding/activity centers with different thermodynamic and kinetic characteristics.

The fact that hydrolysis of esters by albumin, in particular NPA, does not differ significantly from “classical” hydrolysis, was first stated by Casida and Augustinsson in 1959 [13]. Studying the kinetics of hydrolysis of NPA, they found that both of the reaction products, acetate and *p*-nitrophenol, are released in a ratio close to equimolar, and after complete hydrolysis of a single portion of NPA, the enzymatic activity of albumin does not disappear, nor even decrease with the addition of the next batch of substrate. They also showed that the kinetics of the hydrolysis reaction of esters by albumin follows Michaelis-Menten kinetics with the formation of an intermediate complex [48]. In addition, they found that the apparent dissociation constant of the albumin complex with one of the ether substrates studied, *p*-nitrophenyl-*N*-methylcarbamate, is 1.4 × 10^–4^ M, and this value corresponds to the *K_s_* value of many other esterases [13].

The first publication on the existence of at least two different centers (sites) responsible for the two types of activity in the albumin molecule—“true” esterase and pseudo-esterase—appeared in 1972 [14]. In that article, it is noted that the interaction of albumin with NPA has a two-phase pattern: during the first few minutes a “burst” of activity is observed, i.e., there is a rapid formation of the product (mainly nitrophenol), after which the system goes into a stationary mode, with a slower formation of product but not a plateau. The first phase is provided by two processes at two different sites: monoacetylation of albumin as a result of pseudo-esterase activity by one site, and albumin-catalyzed “true” hydrolysis of NPA by the second site. The second phase is due to the activity of the second site only. These two phases are easily reproduced; inhibitory analysis of the first phase using NPA as a substrate was used by Zaidi and co-authors in 2013 [49].

The problems of identification and classification of esterases have been repeatedly discussed by biochemists, especially in the 1970s and 1980s [44,50]. Perhaps like no other group of enzymes, esterases have broad substrate specificity. On the other hand, different enzymes, sometimes belonging to other classes, or not belonging to any class (like albumin, although there are many reasons for its attribution to esterases) can catalyze the same esterase reaction. When discussing the specificity of esterases, the question arises: what is the hydrolytic activity of the enzyme, so that it can be classified as a “true” esterase? For example, α-chymotrypsin and aldehyde dehydrogenase can hydrolyze NPA at a rate (number of turnovers, *k_cat_*) of 0.9 and 54 s^–1^, respectively, but this is certainly too slow compared with carboxyl esterase of the liver hydrolyzing NPA at a rate of 41,000 s^–1^ [44]. Moreover, these enzymes catalyze their specific substrates with much greater efficiency. It has been suggested that an enzyme whose catalytic activity is as high as many thousands of turnovers can be considered a true esterase, but a definite value has not been accepted [50]. It should not however be forgotten that the classic theory of Michaelis-Menten and Briggs-Haldane kinetics [48,51] assumes: (1) an excess of a free substrate (free ligand approximation); (2) stationarity, when d [ES]/dt = 0 (steady state approximation); and (3) the substrate reaches equilibrium on a much faster time-scale than the product is formed or, more precisely, that *k_cat_* << *k*_−1_ and/or *k_cat_* ≤ *k_+_*_1_, i.e., when the values of *K_m_* and *K_s_* are nearly equal (rapid equilibrium approximation). The situation when *k_cat_* >> *k*_−1_ and/or *k*_+1_, or when we do not know *a priori* how many free substrate exist in a stationary state (which is often observed when working with “real” enzymes with a large number of turnovers) means a serious deviation from classical kinetics and requires the use of other models, for example, the van Slyke-Cullen or Morrison models [52,53,54,55]. The former one is an extreme, where *k_cat_* >> *k*_–1_, i.e., the rapid equilibrium approximation is not met; the latter one is a situation where the total enzyme concentration is above the *K*_m_ of the system, i.e., the free ligand approximation is not met (that may well be the case with albumin). Thereby the kinetics of albumin interaction with certain substrates over a certain range of concentrations completely corresponds to classical enzymatic kinetics, perhaps even more than the kinetics of the most “true” enzymes.

Some 60 years ago it was proposed to separate esterases into two groups, A and B [56,57]. Accordingly, B-esterases are inhibited by OPs, carbamates and organosulphur compounds, whereas A-esterases are not inhibited by OPs, but hydrolyze them. A characteristic feature of B-esterases is the presence of serine in the active site; therefore they are also called serine esterases. A-esterases are not well studied but many of them are sensitive to agents that bind sulfhydryl groups, so was suggested that they be called “cysteine esterases” [44]. According to formal characteristics, albumin can be attributed to the group of cysteine A-esterases. This is supported by data on the modulation (pseudo) esterase activity of albumin with malonic dialdehyde and peroxide of linolenic acid [58].

Junge and Krisch in 1973 noted that, from the theoretical point of view, it is very difficult to draw a clear line between esterases that are sensitive to OPs and esterases hydrolyzing OPs because some B-esterases can indeed hydrolyse some OPs (e.g., paraoxon) [44]. The main conclusion of these authors is as follows: there is one principal scheme of the reaction between esterase and ester (Scheme 1), and to consider this or that ester as substrate or inhibitor of esterase (in relation to our problem—to consider the enzyme as “true” esterase or pseudo esterase) depends on the ratio of the rates of formation and hydrolytic cleavage of the covalent complex (adduct).

As for the problems of esterase activity and the possibility of attributing albumin to esterases, we believe that they should be discussed not from the point of the characteristics of A- and B-esterases, but from the point of the existence of several binding/activity sites in albumin with different values of kinetic constants. Thus, some authors [59] consider the hydrolysis of 4-nitrophenyl myristate as pseudo-enzymatic hydrolysis, because deacylation constant *k*_+3_ of the Tyr411 site is much lower than the acylation constant *k_+_*_2_ (*k*_+3_ ~ 10^–5^ s^–1^, *k*_+2_ ~ 0.4 s^–1^). It is important to notice that no other sites have been studied. A comparative analysis of albumin activity towards *para*- and *ortho*-nitrophenyl esters with various side chains as substrates showed that the most “suitable” substrate showing the presence of esterase activity in albumin is *p*-nitrophenylpropionate (Scheme 3): *k_cat_* = 0.166 s^–1^, *K_s_* = 1.55 × 10^–4^ M, *k_cat_*/*K_s_* = 1079 M^–1^s^–1^ [46]. Again, only Tyr411 is the focus of attention. Here it is necessary to note an obscure fact that is not discussed in the overwhelming majority of the articles: for any site of albumin, the first stage of interaction is the formation of a certain ligand-protein complex rather than a covalent adduct, due to relatively weak intermolecular bonds: ionic, dipole-dipole, hydrogen bonds, hydrophobic, van der Waals. As it is practically impossible to determine the constants *k*_+1_ and *k*_−1_ for each individual site separately, one experimental evaluation of the first stage of the interaction of albumin with NPA deserves attention: the formation of a certain “generalized” enzyme-substrate complex, the initial quasi-equilibrium state that takes less than 1.1 ms to achieve. The following kinetic characteristics were obtained (Scheme 2): *k*_+1_ ≥ 6.8 × 10^6^ М^–1^s^–1^; *k*_−1_ ≥ 3.5 × 10^3^ s^–1^; *K*_s_ = (4.7 ± 0.5) × 10^–4^ M [45]. Let us also mention some other constants, given in this article, but referring to a specific Tyr411 site: *k*_+2_ = (4.2 ± 0.4) × 10^–1^ s^–1^; *k*_+2_/*K*_s_ = (8.9 ± 0.9) × 10^2^ М^–1^s^–1^ (22 °C, pH 7.5). Borrowing the missing constant *k*_+3_ = 3.2 × 10^–6^ c^–1^ (at pH 8.0) from reference [25], the authors calculated the Michaelis constant: *K*_m_ = (*K*_s_ × *k*_+3_)/(*k*_+2_ + *k*_+3_) ~ 3 × 10^–9^ M [45]. Such a small *K*_m_ can be obtained if parameters of the generalized equilibrium state of all sites of the albumin molecule interacting with NPA (or another substrate) are attributed to one site, Tyr411. Theoretically, the total number of these sites can reach 82, if we take into account all those amino acid residues that are acetylated by prolonged incubation of albumin with supersaturated concentrations of NPA [25]. In this case, the Michaelis constant should at least be designated as apparent (observed), and the rate constant *k*_2_ instead of *k*_1_ should better be taken for estimation of specific *K*_s_. Continuing the calculations: *k*_+3_/*K*_m_ = 1 × 10^3^ М^–1^s^–1^. To compare, similar parameters calculated in the study of the interaction of acetylcholine with AChE (Scheme 5): *k*_1_ = 6.7 × 10^4^ М^–1^s^–1^, *k*_−1_ = 87.7 s^–1^, *k*_2_ = 44.6 s^–1^ [47]. If *K_m_* is calculated from these data, we get about 2 × 10^–3^ M. With acetylthiocholine as the substrate, close values can be obtained: *K*_m_ = 8 × 10^–4^ М, *k*_1_ = 20.4 × 10^4^ М^–1^s^–1^, *k*_−1_ = 52.0 s^–1^, *k*_2_ = 12.8 s^–1^. The definition of catalytic efficiency: *k*_2_/*K*_m_ = 2 × 10^4^ М^–1^s^–1^. The differences from other data for acetylcholine (*K*_m_ = 9 × 10^–5^ М, *k*_2_/*K*_m_ = 1.6 × 10^8^ М^–1^s^–1^ [42]) are obviously due to different experimental conditions (25 °C, pH 8, ionic strength 0.11 M). If we compare albumin with lysozyme (both have no noticeable differences between *K_m_* and *K_s_*) *k_cat_* is equal to 0.15 c^−1^, *K_m_* is equal to *K_s_* with a maximum value of 9 × 10^–6^ M at pH 5, hence *k*_cat_/*K*_m_ = 1.7 × 10^4^ М^–1^s^–1^ [60]. Thus, the kinetic characteristics of albumin as an enzyme are actually not so different from those of “classical” enzymes. Of course, the fact that albumin hydrolyzes certain substrates at a low rate cannot be ignored, but the fact that human blood plasma (unlike rodent and lagomorph blood plasma) contains no carboxylesterases that carry out this hydrolysis more efficiently should also be taken into account [61]. The amount of “promiscuous” (i.e., with a broad substrate specificity) paraoxonase 1 (PON1) in human plasma is approximately a thousand times lower than the amount of albumin (by weight), and the kinetic characteristics of PON1 with respect to highly toxic nerve agents (soman and others) do not allow serious consideration of this enzyme as a means of their detoxification at toxicologically relevant concentrations. At the same time, considering the huge amount of albumin in the blood plasma and taking into account the equilibrium nature of the interaction of many substrates with albumin (*k*_−1_ >> *k*_+2_), Scheme 2 [45]), the apparent rate constant of the pseudo-first order reaction may not be so small from the physiological point of view.

### 3.2. Albumin and Organophosphates: Molecular Modeling Studies

Interaction of albumin with OPs is a very interesting area of research both from the basic and applied points of view. Nevertheless, research in this field has not been fulfilled to the extent that all the questions are answered concerning binding and enzymatic activities of albumin. In this section, we consider two main problems that were raised in several previous research studies: interaction of albumin with soman, and comparative study of human and bovine serum albumins.

#### 3.2.1. Studies on Interaction of Soman with Albumin

Soman, which is one of the most toxic OPs, is a mixture of four stereoisomers. The pairs of enantiomers of soman (C(+)P(+) and C(−)P(−); C(+)P(−) and C(−)P(+))are present in the synthetic C(±)P(±)-soman in a ratio of 45:55, with an equivalent number of two enantiomers within each pair [62]. The most detailed study of the kinetics of soman interaction with albumin is presented in a paper by Li et al. [26]. Due to the combined use of mass spectrometry methods (MALDI-TOF and tandem quadrupole mass spectrometer), ^31^P-NMR spectroscopy, spectrophotometric determination of fluoride ion, along with molecular modeling and biochemical studies, the authors solved a system of differential equations and determined the following kinetic characteristics of interaction of albumin with soman (the designations of constants correspond to Scheme 4): bimolecular rate constant of phosphonylation of Tyr411 of albumin *k*_p_ = 15 ± 3 M^–1^min^–1^; the constant of spontaneous reactivation of the site Tyr411 *k*_r_ = 0.0044 h^–1^ = 7.3 × 10^–5^ min^–1^; the constant of spontaneous hydrolysis of soman (determined by the release of fluoride) *k*_hо_ = (3.42 ± 0.07) × 10^–3^ min^–1^; and the constant of the so-called “*spontaneous hydrolysis of soman in the presence of albumin*” (also determined by the release of fluoride) *k*_h_ = (7.6 ± 5) × 10^–3^ min^–1^. Thus, if one compares the constants, “*spontaneous hydrolysis of soman in the presence of albumin*” is not much more than 2-fold faster than spontaneous hydrolysis of soman in water solution, but it is 100-fold faster than the reactivation of the site Tyr411. Despite the rather large standard deviation, two orders of magnitude of a difference between the mean values do not give a reason to doubt the reliability of the difference. Some misunderstanding arises from the fact that the scheme is depicted in such a way that albumin does not seem to participate, and the expression *"spontaneous hydrolysis of soman in the presence of albumin"* is rather strange. Furthermore, in the text of the article, the authors have attempted several times to explain the phenomenon they discovered. In Section 3.2.2 they write first that “*albumin itself provides a physicochemical environment that enhances the spontaneous hydrolysis of soman*”, then they suppose that “*catalysis takes place at a different* *site*” of albumin, below they suggest that “*albumin-mediated hydrolysis of soman occurs via a mechanism that does not involve a covalent intermediate*”, and then again assume “*a nonenzymatic, physicochemical interfacial reaction*”, and finally, to their credit, they come to conclusion at the end of the section: “*we cannot rule out the existence of a second active center*”. The authors [26] could not but come to this conclusion for one simple reason: the MALDI-TOF method, which detects long-lived covalent adducts reliably, made it possible to locate only one adduct of albumin with soman, at the Tyr411 residue. Although the authors modelled the interaction of soman with Tyr411, the full potential of molecular modeling, which would have provided insight into a possible alternative mechanism of “true” hydrolysis, was in fact not applied. In one of the later Section 3.2.2, the authors return to the “inexplicable” phenomenon again, emphasizing the importance of enzymatic “*hydrolysis of soman at a second site (k_h_ = 0.0076 min^–1^) with a rate 100 times higher than the turnover at Tyr411*”. Finally, in the very last paragraph of the article and its last Section 3.3, which mainly deals with possible options of the creation of mutant recombinant albumin, the authors admit the possibility that “*the observed catalysis of soman hydrolysis on the albumin surface at a site different from Tyr 411 could also be improved*”. To estimate the degree of the needed enhancement, the last section of paper [26] gives simple but very important calculations.

As indicated above, the bimolecular rate constant for albumin phosphonylation at Tyr411, *k*_p,_ is equal to 15 ± 3 M^–1^min^–1^. Furthermore, if the albumin concentration in plasma is 600 μM, then the apparent first order rate constant of the reaction of soman with albumin (more precisely, with Tyr411 only) is equal to 9 × 10^–3^ min^–1^. At the same time, the total pseudo first order rate constant for the reaction of soman with AChE and BChE is 4.5 min^–1^, i.e., 500 times higher. This value is obtained if the concentration of AChE in human blood is considered to be equal to 2.5 nM, the BChE concentration to be 50 nM, and the ratio *k*_2_/*K*_d_ for both enzymes to be 9 × 10^7^ M^–1^min^–1^. The 500-fold difference is a very serious challenge, but we recall that the authors rely on the stoichiometric properties of the site Tyr411 only, which is very difficult to amplify 500-fold. However, if we consider the properties of the hypothetical second site, which is 100 times faster and catalytically (not stoichiometrically) interacting with the soman, then the task of enhancing these properties by only 5-fold seems to be more feasible.

At the same time, the real possibilities of albumin for detoxifying OPs were proved by the elegant experiments of a Spanish laboratory under the leadership of Vilanova [63,64,65]. The researchers did not work with soman, but with less toxic OPs: paraoxon, chlorpyrifos-oxon, etc. They did not have modern NMR and mass spectrometers, ionometers, etc., but this does not detract from the significance of the results obtained. Moreover, data obtained in vivo are much more valuable than theoretical calculations based on in vitro studies. These authors found that the level of detoxification of paraoxon by albumin in toxicologically relevant concentrations was no less than detoxification by paraoxonase, so in PON1 knockout mice the sensitivity to paraoxon does not differ from control [64]. In this report [64] and especially in the subsequent report by the Spanish researchers [65], the catalytic activity of albumin is unequivocally stated - the kinetic constants for the studied substrates are given. Moreover, in contrast to the work discussed above [26], the authors of [64,65] give the main role of albumin in the detoxification of OPs as catalytic hydrolysis, and the secondary one as stoichiometric interaction with albumin.

**Figure 1:**
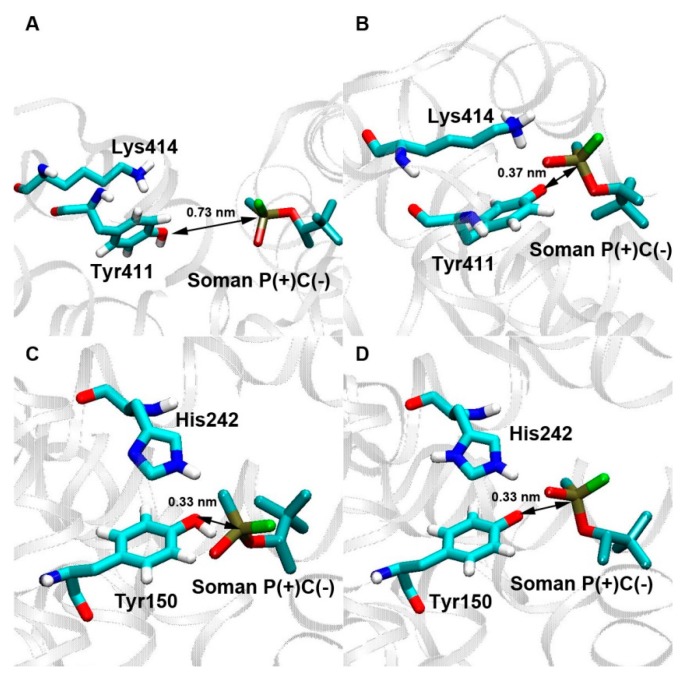
The results of molecular docking of P(+)C(−)-stereoisomer of soman into the sites of albumin Tyr411 (**A**,B) and Tyr150 (**C**,**D**) in the protonated (**A**,**C**) and deprotonated state of the tyrosine (**B**,**D**). In the hypothetically productive complexes (**B**–**D**), the distance between the phosphorus atom of soman and the hydroxyl oxygen of Tyr411 (150) is small enough (<0.4 nm) for the nucleophilic attack of the oxygen [66,67,68]; in addition, fluorine (colored green) and phosphorus atoms of soman and hydroxyl oxygen of Tyr411(150) are positioned in one line as it happens in cholinesterases [69].

Using in silico methods, we have identified all possible sites of interaction between albumin and soman, and the sites of our special interest were those other than Tyr411. The main candidate for the site of esterase activity of albumin was Tyr150. Analysis of the complexes of soman with protonated Tyr411 has shown that dist(P-O) is equal to 0.68 nm for C(+)-isomers, 0.72 and 0.73 nm for P(−)C(−)and P(+)C(−)-isomers, respectively (Figure 1A). It is considered that nucleophilic attack by the catalytic serine hydroxyl of cholinesterases on the carbonyl carbon atom of their natural substrate acetylcholine and other ligands is possible if the distance between them is less than 4 Å [66,67]. Moreover, it was shown earlier that the nucleophilic attack on the phosphorus atom in a substrate can occur if the distance between the phosphorus atom of a ligand and a catalytic atom of an enzyme does not exceed the same value of about 0.4 nm [68]. Taking these data into account we proposed the same criterion for the interaction of albumin with OPs. According to this criterion, the dist(P-O) values that we have obtained are too large for the nucleophilic attack of Tyr411 on the phosphorus atom of soman, and the formation of a new covalent bond between soman and tyrosine is impossible. The values of the dissociation constants are equal to 630 ± 32 μM for P(−)C(−)-isomer, 641 ± 28 μM for P(+)C(−)-isomer, 792 ± 53 μM for P(−)C(+)-isomer, and 919 ± 60 μM for P(+)C(+)-isomer. Stereochemistry of the phosphorus atom in the complexes does not affect the value of *K*_d_ greatly, but C(+) and C(−) stereoisomers interact with the site Tyr411 with different efficiencies. Molecular docking of soman into the site Tyr411 in the non-activated (protonated) state was first made in 2008 [26]. According to the obtained data, the distance between the phosphorus atom of soman and hydroxyl oxygen of tyrosine varied between 0.68 and 0.72 nm, the dissociation constant values were equal to 115–226 μM. The P(+)- and P(−)-stereoisomers of the ligand were sorbed with equal efficiency, the C(+)-enantiomer interacted with the site more weakly than the C(−)-isomer. The results of our calculations of the constants and distances are generally in agreement with the data obtained in the work of Li et al. [26]. The difference in *K*_d_ values can be explained by the fact that in our research the albumin molecule was minimized before the docking procedure, which led to a change in the conformation of the binding sites. In the complexes of soman with deprotonated Tyr411, all the stereoisomers bind at the same position; dist(P-O) is equal to 0.36 nm for the P(+)C(+)-isomer and 0.37 nm for the other three isomers (Figure 1B). In this position, the attack of the hydroxyl oxygen of the tyrosine on the phosphorus atom of soman is possible. The values of *K*_d_ are equal to 466 ± 27 μM for P(−)C(−)-isomer, 524 ± 31 μM for P(+)C(−)-isomer, 425 ± 22 μM for P(−)C(+)-isomer, and 738 ± 44 μM for P(+)C(+)-isomer.

The analysis of the complexes of soman with the protonated Tyr150 has revealed that all stereoisomers bind in the same position; the distance between the hydroxyl oxygen of the tyrosine and the phosphorus atom of soman varies from 0.33 to 0.36 nm (Figure 1C). *K*_d_ values are equal to 280 ± 16 μM for P(−)C(−)-isomer, 496 ± 25 μM for P(+)C(−)-isomer, 192 ± 9 μM for P(−)C(+)-isomer, and 487 ± 26 μM for P(+)C(+)-isomer. *K*_d_ for P(+)-stereoisomers is higher than for P(−)-isomers. These results seem to be very important, given that the P(−)-isomers of soman are more toxic [70]. C(−)- and C(+)-enantiomers bind with the same efficiency. In general, according to our data, soman (including its most toxic enantiomers) bind with the site Tyr150 better than with the site Tyr411. In the complexes of soman with the deprotonated Tyr150 all stereoisomers are also sorbed in the same position; the dist(P-O) value varies from 0.33 to 0.34 nm (Figure 1D). *K*_d_ values are equal to 295 ± 18 μM for P(−)C(−)-isomer, 470 ± 31 μM for P(+)C(−)-isomer, 205 ± 10 μM for P(−)C(+)-isomer, and 457 ± 26 μM for P(+)C(+)-isomer. Again, in such complexes, the P(+)-isomers interact with the site Tyr150 worse than the P(−)-isomers, and the C(−)- and C(+)-enantiomers bind to this site with equal efficiency. Therefore, the protonation state of Tyr150 does not affect the dist(P-O) and *K*_d_ values.

Thus, it has been shown that the formation of the chemical bond between soman and Tyr411 occurs only under the condition of deprotonation of tyrosine. The most important point, however, is that Tyr150 binds soman more efficiently than Tyr411 does. The deprotonation of Tyr150 does not determine the effectiveness of binding, but may affect the stability of the formed complex.

Furthermore, it is known that in productive complexes of cholinesterases with OPs containing CH_3_P(F)(O)O-group, fluorine and phosphorus atoms of a ligand and hydroxyl oxygen of the catalytic serine are positioned in one line [70]. In the complexes of albumin and soman that we consider as productive by the value of dist(P-O), there is the same geometry (Figure 1B–D).

**Figure 2:**
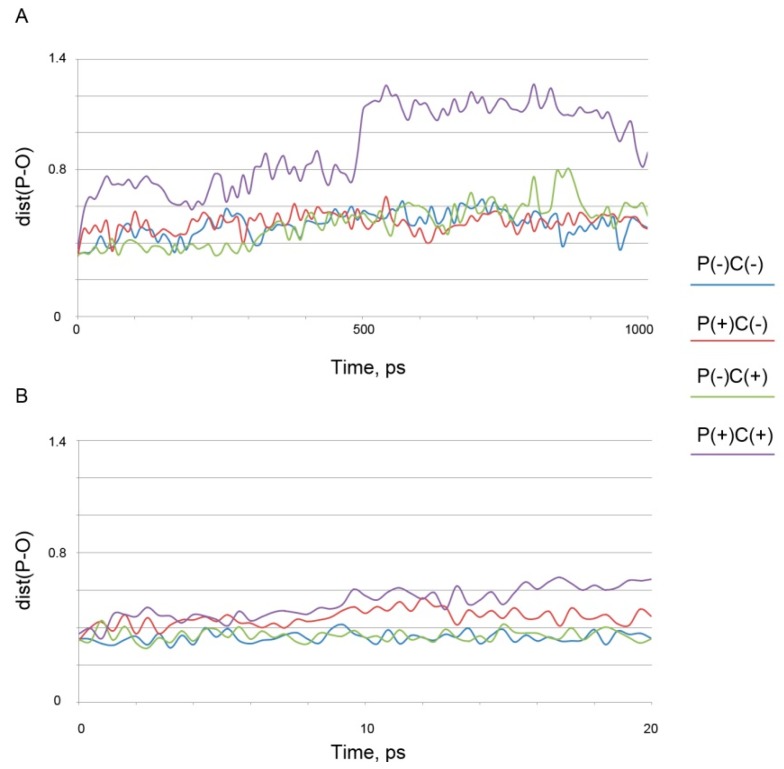
Molecular dynamics simulation time dependence of the distance between the phosphorus atom of soman and the hydroxyl oxygen of Tyr150 (dist(P-O)): the total trajectory (**A**) and the first 20 ps of the simulation (**B**).

We then calculated by molecular dynamics further conformational changes of the ligand-protein complexes. The dependence of dist(P-O) on time were calculated using the trajectories obtained. The analysis of the obtained dependences has shown that the complexes of the stereoisomers of soman with protonated Tyr150 and with Tyr411, in both protonation states, are unstable. During the simulation, the value of dist(P-O) does not descent below 0.38 nm. The results of MD simulation of the complexes of soman with deprotonated Tyr150 differ for different isomers of the ligand (Figure 2A,B).

The complexes of albumin with the P(+)-isomers of soman are unstable, the dist(P-O) value does not descent below 0.38 nm during the simulation, whereas the complexes with P(−)-stereoisomers of soman are stable during the first 20 ps of simulation. It is known that 10 ps is sufficient for the formation of a new covalent bond [71], consequently, albumin complexes with the P(−)-stereoisomers of soman are stable enough for possible covalent bond formation. We then demonstrated that other amino acid residues of albumin can also serve as a site(s) of soman hydrolysis. Earlier, it was shown that 82 amino acids of the albumin molecule could be acetylated by NPA [25]. In our study, the environment of these amino acids, as well as other possible phosphonylation sites (all other serines, lysines, threonines and tyrosines of albumin molecule) were analyzed for the presence of possible proton acceptors that could work similarly to the histidine of the catalytic triad of acetylcholinesterase. Consequently, 40 sites were selected for further verification (11 lysines: 12, 181, 225, 233, 240, 262, 274, 276, 313, 317, 402; 12 serines: 65, 83, 192, 193, 202, 270, 273, 287, 312, 435, 454, 474; nine threonines: 68, 76, 79, 125, 243, 420, 467, 527, 540; 8 tyrosines: 84, 148, 161, 263, 319, 334, 341, 370). Molecular docking of the P(−)C(−)-isomer of soman (as the most toxic stereoisomer) to these phosphonylation sites was carried out. For all the complexes obtained, the values of *K*_d_ and the distances between the phosphorus atoms of soman and the carboxyl oxygen atom (nitrogen in the case of lysine) of a studied amino acid (dist(P-O(N)) were calculated. *K*_d_ of most complexes have been found to be very high (1010–23,100 μM). Only a few values of *K*_d_ are close to those obtained for the sites Tyr150 and Tyr411 (Table 1).

The result of the molecular docking of soman into the site Ser192 is a complex with *K*_d_ = 581 ± 10 μM. However, the distance between the phosphorus atom of the ligand and the oxygen of the hydroxyl group of the serine is equal to 1.1 nm. At the same time, the graphical analysis has revealed that soman binds near Ser193; the distance between the phosphorus atom of the ligand and the hydroxyl oxygen of Ser193 is only 0.38 nm. The result of the molecular docking of soman into the site Ser193 is a complex with *K*_d_ = 518 ± 14 μM. The resulting conformation coincides with the result of docking into the site Ser192.

Thr243 was also not indicated as an acetylation site in the report by Lockridge et al. [25]. The analysis has shown that molecular docking of soman into the site Thr243 results is a complex with *K*_d_ = 290 ± 6 μM. However, the distance between the oxygen atom of the hydroxyl group of the threonine and the phosphorus atom of soman is equal to 1.28 nm. Graphical analysis has revealed that in this complex, soman binds in the site Tyr150; the distance between the phosphorus atom of soman and the hydroxyl oxygen of the tyrosine is equal to 0.35 nm. The resulting conformation is consistent with the result of the docking of soman into the site Tyr150. The result of the molecular docking of soman into the site Ser287 is a complex with *K*_d_ = 295 ± 15 μM, while the distance between the hydroxyl oxygen of the serine and the phosphorus atom of soman is 0.77 nm. The analysis has shown that soman is again sorbed in the site Tyr150, with the distance between the phosphorus atom of soman and the hydroxyl of the tyrosine being 0.35 nm. The resulting conformation is also consistent with the result of the docking into the sites Tyr150 and Thr243. The graphical analysis of the complex of soman with the site Lys402 has shown that the ligand binds near the lysine residue. *K*_d_ of the complex is 737 ± 27 μM, and the distance between the phosphorus atom of the ligand and the Nζ atom of the lysine is 0.37 nm. *K*_d_ of the complex of soman and the site Ser454 is equal to 572 ± 17 μM. However, the distance between the hydroxyl oxygen of the amino acid and the phosphorus atom of soman is 1.14 nm. The graphical analysis has revealed that, in this case, soman binds in the site Tyr411; the distance between the phosphorus atom of soman and hydroxyl oxygen of the tyrosine is 0.71 nm. The conformation obtained coincides with the result of the docking of soman into the protonated site Tyr411. The result of the molecular docking of soman into the site Thr540 is a complex *K*_d_ = 759 ± 21 μM. The graphical analysis has shown that soman binds near Lys402; the distance between the phosphorus atom of soman and the atom Nζ of the lysine is 0.37 nm whereas the distance between the hydroxyl oxygen of Thr540 and the phosphorus atom of soman is 0.87 nm. The conformation that we obtained coincides with the result of the docking of soman near Lys402. According to the authors of [25], the serines 192, 287, 454 and Thr540 of albumin are acetylated by NPA. The complexes of soman with these sites have not been identified in our study. We suppose that the energies of these complexes are much higher than the energy values of the complexes of soman with the sites Ser193, Tyr150 and Tyr411, so it was not possible to obtain them by molecular docking method. It is important to emphasize here that the adducts of NPA with Ser193 were not detected by the authors of [25]. The absence of the stable covalent adducts under the conditions favorable for the formation of a stable complex (dist(P-O) = 0.38 nm, *K*_d_ = 518 ± 14 μM) indicates that Ser193 can provide true esterase activity of albumin and hydrolysis of soman molecule. It is important that near Ser193 there is a histidine residue, and the distance between the hydroxyl oxygen of the serine and the atom Nδ of the histidine, which can accept the proton from the serine, is only 0.22 nm.

Thus, according to the docking data, in addition to the sites Tyr150 and Tyr411, the sites Ser193 and Lys402 can interact with soman productively. To investigate the properties of these sites, the remaining P(+)C(−)-, P(−)C(+)- and P(+)C(+)-stereoisomers of soman were docked into these binding sites and the results (*K*_d_ and dist(P-O(N)) are presented in Table 2. In the case of Ser193 and in the case of Lys402, only the P(−)-isomers of the ligand bind to these sites at the distance sufficiently close for the nucleophilic attack on the phosphorus atom of soman.

To verify the accuracy of the calculations and the suitability of the method used, a kind of positive control was applied: we carried out the molecular docking of NPA into the sites Ser193 and Lys402. The analysis of the obtained complexes has shown that in the case of Lys402, *K*_d_ is equal to 27 ± 2 μM. The distance between the carbonyl carbon of the ligand and the atom Nζ of the lysine is 0.37 nm and is sufficient to form a new covalent bond between NPA and the lysine residue. In the case of Ser193, *K*_d_ is equal to 40 ± 5μM. However, the graphical analysis has shown that the ligand binds at the distance of 1.4 nm from the serine residue, so that the attack of the serine on the carbonyl carbon of NPA is impossible. The analysis of the ligand environment in this complex has shown that NPA is sorbed in the region of Ser454, which can be acetylated. Thus, our data on molecular docking agree with the results of Lockridge et al. [25]. The deprotonation of catalytic tyrosine is necessary for the productive binding of soman in the site Tyr411. The activation of tyrosine affects the geometry of the binding of soman in the site Tyr150 much less, but improves the stability of the complexes. The processes leading to the transfer of the proton from the tyrosines to the acceptors in the albumin molecule remain unclear. Perhaps the transfer of the proton is regulated by the binding of ligands in other sites of albumin (allosteric modulation).

In addition, we have hypothesized the involvement of not only Arg410 but also Lys414 in the site Tyr411 and His242 in the site Tyr150 as proton acceptors from the tyrosine residues. Thus, it is possible that the hydrolysis of some substrates by albumin requires a catalytic dyad His-Tyr or Arg(Lys)-Tyr, in which histidine or lysine residues act as an acid residue; such cases are described in the literature but with the difference that two different histidine residues serve as acid and basic residues [72]. It can be assumed that in the pair of two main sites interacting with NPA and OPs, the Sudlow I site with the Tyr150 residue exhibits the "true" esterase activity, and the Sudlow II site with the Tyr411 residue—the pseudo-esterase. Such separation may prove to be rather conditional if it is revealed that in both cases an intermediate covalent adduct is formed, and the differences are in the lifetime of this adduct, i.e., the difference consists in the constants *k*_2_ and/or *k*_3_, which characterize the acylation and deacylation reactions, correspondingly. If this is so, the result of the reaction in this site should be an enzyme (albumin) and two products of hydrolysis of the substrate (fluoride and pinacolyl methylphosphonic acid, PMPA) and it is impossible to detect the intermediate adduct by MALDI mass spectrometry. To confirm these assumptions, it is necessary to investigate the kinetics of the formation of PMPA, and to conduct additional in silico calculations, which will help to study the interaction of albumin with OPs and other ethers in more detail. This research has not just theoretical, but also great practical significance. Understanding of the mechanisms of binding and esterase activity of albumin will allow the targeted regulation (modulation) of the toxic or pharmacological properties of many biologically active substances.

#### 3.2.2. Comparative Analysis In Silico of Paraoxon Binding with Human and Bovine Serum Albumin

In in vitro experiments devoted to studying the (pseudo-)esterase activity of albumin, both human [26,73] (HSA) and bovine [74,75] serum albumin (BSA) are used. The primary sequences of HSA and BSA have 76% homology [76]; Tyr410 and Tyr149 in BSA molecule correspond to Tyr411 and Tyr150 in HSA molecule [76]. Nevertheless, there are a plethora of data to indicate that these two proteins differ in their effectiveness of interaction with a range of xenobiotics. Thus, a comparative analysis showed that HSA more efficiently binds caprofen and 2-anthracene-carboxylic acid [77]. It has been shown [78] that blue dextran binds to HSA only and does not interact with BSA. In other work [79], it has been shown that warfarin interacts more efficiently with BSA than with HSA, while contrary to this its structural analogue acenocoumarol, which contains an additional NO_2_ group, interacts more effectively binds to HSA. The polyphenols curcumin and diacetylcurcumin interact much more efficiently with BSA than with HSA [80]. It was found that pyridoxal phosphate binds ten times less efficiently with BSA than with HSA [81], and methyl parathion 1.5 times better interacts with HSA than with BSA [82]. Thus, a cursory analysis of the literature data does not reveal any patterns from which it is possible to determine *a priori* whether the OPs will better interact with HSA or BSA. We suggested that such patterns could be identified using molecular modeling methods, which enables one to determine and compare the structural characteristics of the binding sites of these enzymes responsible for the effectiveness of their interaction with ligands. This at least would help to carry out a comparative analysis of these proteins from an evolutionary point of view.

As a first stage, molecular docking of paraoxon into the sites Tyr150 (149) and Tyr411 (410) was performed. Then the conformational changes of the obtained complexes were calculated using 10 ns MD simulation. The approach and the parameters of the docking and MD procedures are described in the Methods section. Based on the results of the docking procedure, the dissociation constants of the ligand-protein complexes (*K*_d_) and the distances between the phosphorus atom of paraoxon and the hydroxyl oxygen of the tyrosines (dist(P-O)) were estimated. The complexes with the minimal value of dist(P-O) were selected for further analysis. Thus, the model-1 was selected for the complex of paraoxon with the site Tyr150 of HSA; model-2—for the complex of paraoxon with the site Tyr411 of HSA; model-5—for the complexes of paraoxon with the sites Tyr149 and Ty410 of BSA. Energetic and geometric characteristics of these complexes are given in Table 3.

**Figure 3:**
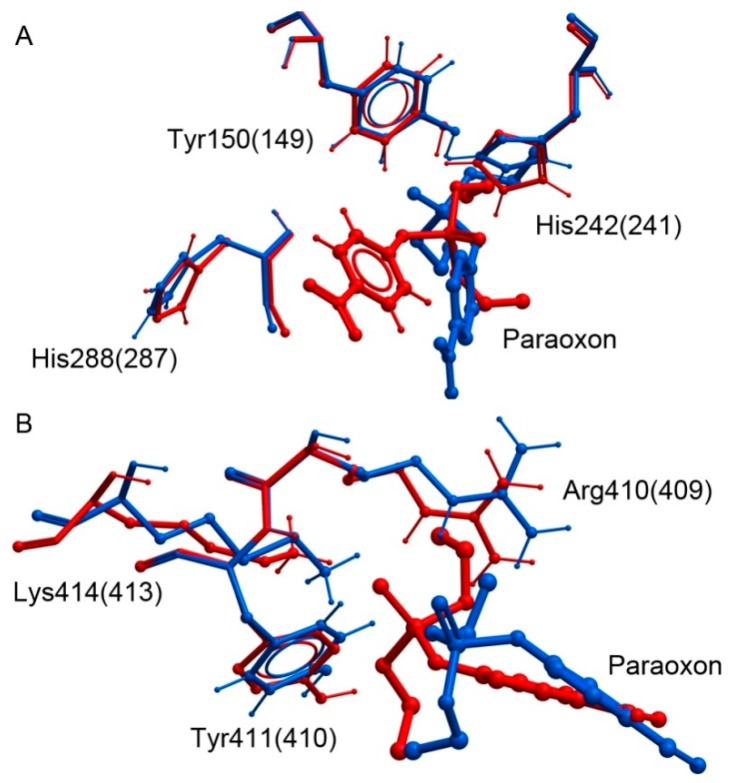
The result of molecular docking of paraoxon into the sites Tyr150 (149) (**A**) and Tyr411 (410) (**B**) of human (blue) and bovine (red) serum albumin.

Figure 3A shows the results of molecular docking of paraoxon into the sites Tyr150 of HSA and intoTyr149 of BSA. Despite the values of *K*_d_ being similar, the position of paraoxon inside the binding sites for these two proteins differs significantly in the orientation of the NO_2_-group and, as a consequence, in the orientation of the phosphoryl oxygen atom of the ligand relatively to the tyrosine residue.

Analysis of the paraoxon environment has revealed that in the case of HSA, the NO_2_-group of the ligand forms two hydrogen bonds with the NH_2_-groups of Arg222. In the BSA molecule, there is a lysine residue in this position, which can form only one hydrogen bond. In the case of BSA, the NO_2_-group of paraoxon forms two hydrogen bonds with the NH_2_-groups of Arg198; in the HSA molecule, this position contains a lysine residue. The result agrees with a previously published report [83], which shows that the difference in the binding efficiency of ergosterol with HSA and BSA can be due to the formation of a hydrogen bond between ergosterol and Arg198. The formation of two hydrogen bonds has a stronger effect on the strength of a ligand-protein complex (and, therefore, on the decrease of *K*_d_) than one hydrogen bond. Therefore, in the most energetically favorable conformations of the complexes of paraoxon with albumin obtained by docking, the NO_2_-group of the ligand interacts with the nearest arginine residue. The different position of the nearest arginine in HSA and BSA also explains the different position of paraoxon in the sites Tyr150 of HSA and Tyr149 of BSA, though the values of *K*_d_ are similar. Figure 3B shows the results of molecular docking of paraoxon into the sites Tyr411 of HSA and Tyr410 of BSA. Despite the significant difference in the estimated values of *K*_d_, the position of paraoxon is similar for HSA and BSA. The comparative analysis of the energy components showed that the contribution of the electrostatic interactions between the protein and the ligand is rather similar for both proteins, while the difference is due to the energies of the hydrogen bonding and van der Waals interactions. Such difference in these characteristics can be explained by the difference in the orientation of the side chains of the amino acids of the site, mainly the position of the side chain of Lys414 (413). The paraoxon molecule is closer to the lysine in BSA than in HSA, and an additional hydrogen bond between the phosphoryl oxygen atom of paraoxon and NH_3_-group of the lysine is formed.

**Figure 4:**
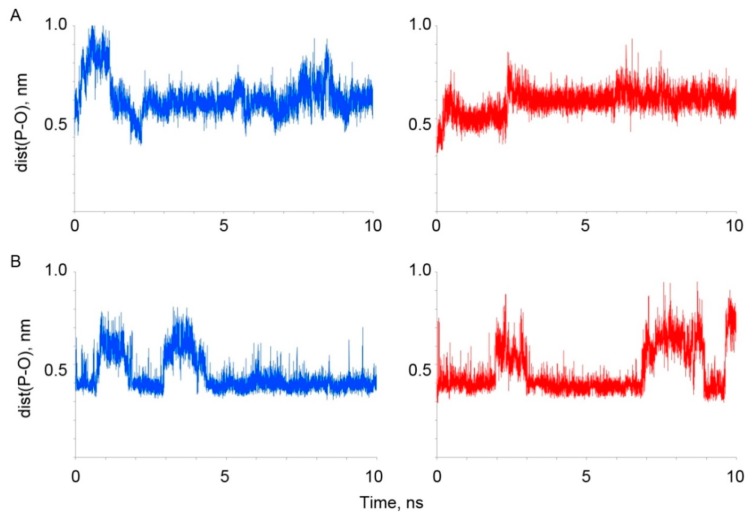
Time dependence of the distance between the phosphorus atom of paraoxon and the hydroxyl atom of Tyr150 (149) (**A**) and Tyr411 (410) (**B**) of HSA (blue) and BSA (red).

The stability and conformational changes of four selected albumin-paraoxon complexes were studied by 10 ns MD simulation. The dependence of the RMSD values of the amino acid residues of albumin on time was calculated. For all complexes, the RMSD value of amino acid residues increased during the first 3 ns of the simulation, then stayed on the level of 0.4–0.5 nm, which indicates the stabilization of the polypeptide chain of the albumin molecule. The exception was the complex of paraoxon with the site Tyr410 of BSA. For this complex, the RMSD value reached the plateau after 8 ns of simulation. The dependence of the values of dist(P-O) on time was also calculated. The results are shown in Figure 4.

The analysis of the data obtained has shown that the complex of paraoxon with the site Tyr150 of HSA is unstable. During the first 2 ns of the simulation, the value of dist(P-O) oscillates in the interval of 0.4–1.0 nm. During the last 8 ns of the simulation it remains unchanged and fluctuates insignificantly at the level of 0.6 nm. The analysis of the position of paraoxon in this part of the trajectory has shown that paraoxon is fixed by the Coulomb interaction between the phosphoryl oxygen of the ligand and the side chain of Arg257. It should be noted that in BSA, there is an arginine in this position too.

According to the data of Lockridge et al. [25], there are 82 amino acids that can be acetylated by NPA, which means that they can theoretically interact with OPs. It was shown earlier that the nucleophilic attack on the phosphorus atom in a substrate can occur if the distance between the phosphorus atom of a ligand and a catalytic atom of an enzyme does not exceed 0.4 nm [68]. Therefore, the analysis of the environment of the phosphorus atom of paraoxon was made. It has revealed that within this distance, there are no amino acids capable of forming a covalent bond with the ligand. The complex of paraoxon and the site Tyr149 of BSA is also unstable (Figure 4A). The value of dist(P-O) increases to the level of 0.6 nm for the first 2 ns and then remains unchanged during the last 8 ns of the simulation. The analysis of the position of paraoxon in this part of the trajectory has shown that paraoxon is fixed by the hydrogen bond between the phosphoryl oxygen of the ligand and the atom Hε2 of His241, and by the Coulomb interaction between the NO_2_-group of paraoxon and the side chain of Gln195. In HSA, these positions have a histidine and a glutamine too. The analysis of the environment of paraoxon in this complex has revealed that within 0.4 nm of the phosphorus atom of the ligand, there are no amino acids capable of forming a covalent bond with a paraoxon molecule.

Thus, the stable position of paraoxon in the sites Tyr150 of HSA and Tyr149 of BSA is different for these proteins, despite the conservativeness of the environment of the catalytic tyrosine. The graphical analysis has revealed that in the case of BSA, the amino acids of the site Tyr149 are positioned more compactly, which makes the steric size of this binding site smaller in BSA than in HSA. However, for both HSA and BSA, the formation of a covalent bond between paraoxon and Tyr150 (149) is impossible in an unmodified protein. According to our earlier data, the trigger that improves the affinity of the site Tyr150 (149) for OPs can be either the transfer of a proton from a catalytic tyrosine to the imidazole ring of His242(241) [84], or the site modulation by endogenous ligands, for example, by fatty acids [85].

The time dependence of the value of dist(P-O) for the complex of paraoxon and the site Tyr411(410) is shown in Figure 4B. The dynamics of this dependence is similar for HSA and BSA: areas of “stability”, when the distance varies within 0.37–0.40 nm, alternate with the areas of “instability”, when paraoxon moves away from the catalytic tyrosine. The graphical analysis has shown that in all "stable" regions, for both HSA and BSA, the position of paraoxon is the same: the ligand molecule is stabilized by the formation of a hydrogen bond between the tyrosine OH-group and the phosphoryl oxygen atom of paraoxon. The analysis of the distance between the hydroxyl hydrogen of Tyr411 (410) and the phosphoryl oxygen of the ligand has shown that the periods of “stability” coincide with the periods of existence of the hydrogen bond between these atoms. Moreover, an increase in the distance between paraoxon and the tyrosine is preceded by the rupture of this hydrogen bond. For both proteins, there are regions of “stability” in which the distance between paraoxon and the hydroxyl oxygen atom of the catalytic tyrosine is sufficiently close to form a covalent bond between the ligand and the protein [68].

Thus, according to the data obtained, the site Tyr411 (410) is more conservative than the site Tyr150 (149): amino acids taking part in the binding of paraoxon in this site (Tyr411 (410), Arg410 (409), Lys414 (413), Arg257 (256)) are the same for both albumins. For both HSA and BSA, the site Tyr411(410) is the primary site of phosphorylation: additional conditions (binding of modulators at other sites) are not required for the productive binding of an OPs molecules and, according to the data of MD simulation, the mechanism of the phosphorylation reaction is the same for both proteins. These data agree with the data of our previous studies [84,85,86,87] and with in vitro experiments [88,26]. Therefore, for irreversible binding of the OPs without additional modulators, we can expect similar results for HSA and BSA. It should be noted that in the case of interaction of albumin with soman, the productive sorption of a soman molecule in the site Tyr411 and its further phosphonylation needs the deprotonation of Tyr411 (see Section 3.2.1). We suppose that it could be due to the different position of phosphoryl oxygen inside the site during the processes of phosphonylation and phosphorylation, like it occurs in cholinesterases [70]. The Tyr150 (149) site is less conservative. In both proteins, the arginine closest to the catalytic tyrosine forms two hydrogen bonds with the ligand molecule and thus participates in the binding of paraoxon. In the HSA molecule, it is Arg222, in the BSA molecule—Arg198. These arginines are located at the opposite sides of Tyr150 (149); therefore, for rigid molecules or for optically active compounds, different binding efficiencies in this site can be expected. According to the data on molecular docking, the energy characteristics of the binding of paraoxon with the site Tyr150 (149) are better than those for the site Tyr411 (410), especially for HSA. On the other hand, according to the data on molecular dynamics, the conformation of the complex of paraoxon-Tyr150 (149) is not energetically favorable. For the stabilization of this conformation, probably some changes are needed for both HSA and BSA structures: either proton transfer from the catalytic tyrosine residue to the imidazole ring of the neighboring histidine His242 (241), or conformational changes caused by ligand binding in other sites. In the stable complexes of albumin with paraoxon non-productively bound in the site Tyr150 (149), the position of the side chains of the nearest amino acids is different for HSA and BSA. This may be due to the different charge distribution in these proteins: the total charge of the HSA molecule is -14e, and for BSA it is -16e. It was previously shown [6] that in HSA, the single Cys34 is oxidized in a part of the albumin molecules. Oxidation of cysteine can also affect the distribution of charges within the albumin molecule, and hence the conformation of the site Tyr150 (149) and the binding efficiency of the molecules in this site. The modulation mechanism of the site Tyr150 (149) requires further research.

### 3.3. Substrate Specificity of Albumin and Potential for its Assignment to Enzyme Nomenclature

Several years ago, Dr. Kragh-Hansen made a fruitful attempt to summarize available data on enzymatic activities of albumin [89]. As a redox-modulating agent, albumin exhibits thioesterase [90,91], glutathione peroxidase and cysteine peroxidase activities [92,29], as well as peroxidase activity towards lipid hydroperoxides [29,30,92,93]. Two cysteine residues, Cys392 and Cys438, complexed with palmitoyl-CoA play an important role as they form a redox active disulfide. Albumin is also a scavenger for active radicals, due to a Cys34 thiol group and six methionine residues [94,95]. The *N*-terminal residue of human albumin, Asp-Ala-His-Lys, complexed with Cu^2+^ ions possesses significant superoxide dismutase activity [96]. Albumin stoichiometrically inactivates hydrogen peroxide and peroxynitrite due to reversible oxidation of Cys34 to sulfenic acid derivative [93]. Cyanide detoxification reaction that yields thiocyanate and is catalyzed by subdomain IIIA residues (excluding Tyr411) is also related to this group of activities. Finally, pro-oxidant properties of albumin should also be noted: albumin-bound Cu^2+^ ions enhance the buildup of ascorbate radical, while molecular oxygen and protons oxidize Cu^+^ ions back to Cu^2+^ [97].

Cysteine peroxidase activity of albumin towards phospholipid hydroperoxides is not high enough (and its glutathione, cysteinyl glycine and homocysteine peroxidase activitiesare obviously even lower), but it was objectively noted [29] that such low activity is compensated by its high blood concentration. To this end, cysteine is a major low-molecular blood thiol that has a physiological concentration of 9–12 µM [98] and the overall concentration of phosphatidylcholine hydroperoxides in plasma is 20–430 nM [29]. Albumin probably contributes to reduction of phospholipid hydroperoxides in plasma together with other peroxidases. At the very least, albumin definitely possesses cysteine peroxidase activity (cysteine:phospholipidhydroperoxide oxidoreductase). Monomeric (but multidomain) albumin does not bind selenium ions unlike anintracellular monomeric Se-bound phospholipid hydroperoxide glutathione peroxidase (glutathione peroxidase 4, PHGPx, GPx4, EC 1.11.1.12), which plays an important role in cellular protection against the damaging effects of lipid hydroperoxides [99,100,101], and also unlike an extracellular tetrameric glutathione peroxidase 3 (GPx3, EC 1.11.1.9), a decreased activity of which is associated with enhanced risk of cancer diseases. With this background, we propose the inclusion of albumin into Enzyme Nomenclature with the number EC 1.11.1.24 [102]. Concerning the physiological role of albumin in susceptibility and pathogenesis of various diseases, a paper by Fischer and co-authors [103] presents evidence of a tight association between albumin concentration (its enzymatic activity was not studied, unfortunately) and the levels of orosomucoid andcitrate, the size of very low density lipoproteins (VLDL) and the risk of fatal outcome, regardless of the kind of chronic disease.

Prostaglandin-D synthase and other types of albumin activities, related to prostanoid metabolism, should also be mentioned: for instance, 15-keto PGE_2_ is dehydrated by Arg257 site yielding 15-keto PGA_2_. Albumin also exhibits exotic activities, such as glucuronidase activity (for instance, (*S*)-carprofen glucuronide—an NSAID—hydrolysis mediated by tyrosine and lysine residues) [18,104,105], and enolase activity [106,107]; the latter is highly important for differential diagnosis of benign and malignant tumors.

Most studies over several decades are related to two groups of albumin activities. One group is comprised of carboxylesterase (EC 3.1.1.1), arylesterase (EC 3.1.1.2), and arylacylamidase activities (EC 3.5.1.13) [22,23]. It is worth mentioning that the last of these was studied using two different substrates, *o*-nitroacetanilide and *o*-nitrotrifluoroacetanilide, and it was shown that hydrolysis yielding *o*-nitroaniline and acetate/trifluoroacetate is mediated by Tyr411. Moreover, both reaction products do not form covalent adducts with albumin, being released simultaneously to the medium. Contrary to the above, acetylsalicylate deacetylase activity (EC 3.1.1.55) [108] may be just a form of pseudoesterase activity [109].

The other group comprises various phosphatase related activities: phosphomonoesterase (EC 3.1.3.2) [16], RNA-hydrolase or phosphodiesterase activity (EC 3.1.4.16), and phosphotriesterase activities (EC 3.1.8.1, EC 3.1.8.2) [23,65]. Aryldialkylphosphatase (EC 3.1.8.1) and diisopropyl-fluorophosphatase (EC 3.1.8.2) fall within subsubclass 3.1.8 (phosphoric triester hydrolases) [110,111]. Aryldialkylphosphatase is commonly known as paraoxonase. Its other names are A-esterase, aryltriphosphatase, esterase B1, esterase E4, pirimiphos-methyl-oxon esterase, paraoxon hydrolase, and aryltriphosphatedialkylphosphohydrolase. It hydrolyzes esters of tribasic phosphoric, two-basic phosphonic, and monobasic phosphinic acids. Its characteristic feature is that the enzyme is inhibited by chelating agents, as divalent ions (mainly, Ca^2+^) are required for the reaction [112]. Carbarylase (esterase E4) which hydrolyzes carbamates is also apparently identical to aryldialkylphosphatase [113]. Diisopropylfluorophosphatase (other names—DFPase, tabunase, somanase, organophosphateanhydrolase, organophosphate anhydrase, diisopropylphosphofluoridase, dialkylfluorophosphatase, isopropyl phosphofluoridase, diisopropylfluoro-phosphatedehalogenase, diisopropylfluorophosphatefluorohydrolase) breaks anhydride bonds of phosphorus (phosphorus halides and phosphorus cyanides) even in warfare organophosphorus compounds (tabun, sarin, soman). Its enzyme activity, similar to aryldialkylphosphatase (EC 3.1.8.1), depends on divalent ions, EC 3.1.8.2 was assigned to this enzyme in 1992 mostly based on 1950s studies, and just one paper dates from 1989 [114,115,116,117,118,119]. Recent data on both enzymes and their dependence on Ca^2+^ ions suggest that diisopropylfluorophosphatase and aryldialkylphosphatase are different names for the same enzyme, which is commonly known as paraoxonase 1 (PON1) [120,121]. However, this name leads to a misconception that paraoxon is the best substrate for this enzyme, albeit the fact that the latter hydrolyses phenylacetate 1000 times faster than paraoxon [122]. The physiological role of PON1 is supposedly hydrolysis of homocysteine lactone, which prevents homocysteinilation of proteins and development of atherosclerosis [123,124]. Furlong and co-authors proved that paraoxon and phenylacetate are both hydrolysed by the same enzyme, and thereby ended long debates on paraoxonase substrate specificity [125,126]. Thus, PON1 acts both as arylesterase (EC 3.1.1.2) and aryldialkylphosphatase (EC 3.1.8.1). Residues involved in phosphotriesterase and esterase/lactonase activities of PON1 are in different loci of its active site [127]. Phosphotriesterase and esterase/lactonase activities are so closely coupled that both are considered as an integral parameter of blood biochemical analysis in recent studies [128,129,130].

It is worthwhile to quote from 25 years ago, the relevance of which has not diminished during this time: *«The characterization and classification of the two groups of esterase discussed here is hindered by a common problem - the shortage of pure preparations of the enzymes»* [131]. Thus, one and the same enzyme probably acts as aryldialkylphosphatase (EC 3.1.8.1), diisopropylfluorophosphatase (EC 3.1.8.2), arylesterase (EC 3.1.1.2), and lactonase (EC 3.1.1.25). It was shown that albumin functions as a paraoxonase though does not depend on Ca^2+^ ions; this fact is recognized in differential analysis of these enzymatic activities [63,64,65,132].

In 1986, concerns were raised over the adequacy of esterase nomenclature and albumin was referred to as a protein that acted as an esterase, but was not assigned an EC number [133]. Recent studies draw attention to classification issues and urge us to submit new data on enzymatic activity of proteins to improve current enzyme nomenclature [134].In accordance with the classification of esterases hydrolyzing organophosphates [131], and considering the revealed features of albumin, this protein may be assigned to two groups (sub-subclasses): carboxylic ester hydrolases (EC 3.1.1), and phosphoric triester hydrolases (EC 3.1.8). A broad substrate specificity and independence of Ca^2+^ ions do not allow us to assign albumin to any established number in enzyme nomenclature. Thus, its place is yet to be defined, and now we suggest at least two numbers as a draft assignment: EC 3.1.1.103 and EC 3.1.8.3 [102]. These numbers would be complementary to the proposed number in the oxidoreductases class, EC 1.11.1.24.

## 4. Perspectives

Understanding of the binding and catalytic possibilities of albumin allowed us to identify two problems related to albumin within toxicological studies and translational medicine. The problem of poisoning by OPs (dealing with organophosphorus insecticides and pesticides, disposing chemical weapons, undergoing poisonous agents) remains relevant, and it is possible that albumin due to its large amount in blood plasma can act as the main modulator of OPs activity in toxicologically relevant concentrations and doses. Therefore, the study of the interaction of OPs with albumin is important from both fundamental and practical perspectives. Albumin has at least two sites of interaction with OPs. In one (Sudlow II), a covalent adduct is formed. In the other (Sudlow I) the covalent adduct is not formed, but it catalytically interacts with the OPs. The problem is that both the formation of the covalent adduct with OPs and catalytic cleavage of the OPs occur slowly, therefore albumin primarily serves as a “vehicle” for the delivery of OPs to the sites with much higher affinity (cholinesterases). For therapeutic purposes, several strategies are proposed to be applied either individually or concurrently: (1) to promote the formation of the covalent adduct in the site Sudlow II (which would lead to detoxification of the OPs); (2) to strengthen the binding of albumin to the OPs and to accelerate the hydrolysis in the site Sudlow I; (3) to prevent “the passenger” from “landing the vehicle”, and then an OP molecule (“the passenger of albumin”) would rapidly find and irreversibly bind to blood cholinesterases, which would be better than the same binding outside the bloodstream especially AChE of neuromuscular and neuronal synapses. This could be done with the help of endogenous or exogenous ligands that modulate the binding and esterase properties of albumin.

Another range of the problems is the possibility of molecular modeling when studying the interaction of albumin with OPs and the translation of the obtained data to the human organism. Experiments in vivo, which are most closely approximated to actual poisoning situations, are conducted mainly in rodents; the use of other mammals for research is rare and difficult nowadays. Some studies showed that human (HSA) and rat (RSA) serum albumins have similar binding characteristics for biologically active substances [135,136]. However, there is evidence that the effectiveness of albumin interaction with some xenobiotics varies for HSA and RSA [137,138]. Therefore, for the correct extrapolation of the results obtained in vivo in rats to the human organism, it is necessary to identify amino acids involved in the interaction of albumin with OPs, to determine all the structural and conformational features of the sites of binding of toxic agents, to compare the characteristics obtained for HSA and RSA. This analysis is possible only by in silico methods. In silico comparison of the effect of OPs and other toxic and medical agents on rat and human albumin will facilitate prediction of how a compound will behave in a pre-clinical experiment in rats and how its action will differ in a human subject. In silico experiments require the three-dimensional model of a protein. Usually the 3D-structures obtained by X-ray analysis and stored in the PDB are used [34]. In the absence of X-ray data, the three-dimensional structure of a protein can be obtained by homology modeling, which is the construction of a 3D model of the protein using its primary sequence and the known three-dimensional structures of homologous proteins [139]. The X-ray analysis of rat albumin has not been carried out yet; therefore, one of the main tasks of molecular toxicologists should be the construction of a three-dimensional model of RSA, the improvement of its geometry, and the analysis of the reliability of the resulting structure. The only way to do it is homology modeling based on the primary structure of RSA and resolved 3D-structures of albumins of other organisms.

Thus, comparative and modeling studies, together with a new therapeutic strategy—interference to the traffic of OPs with albumin—are the perspectives which we rely upon in our studies, bearing in mind that many types of its natural “passengers” (e.g., nutraceuticals) can help the organism to trip safely through life.

## Figures and Tables

**Table 1:** The results of molecular docking of the P(-)C(-)-isomer of soman into the possible sites of phosphonylation or esterase activity of albumin.

Binding Site	*K*_d_, μM	Dist (P-O(N)), nm	Amino Acid Closest to the Ligand	Distance to the Closest Amino Acid, nm	Acetylation by *p*-Nitrophenyl Acetate [25]
Tyr411	601 ± 26	0.72	-	-	+
Tyr150	280 ± 3	0.36	-	-	-
Ser192	581 ± 10	1.06	Ser193	0.38	+
Ser193	518 ± 14	0.38	-	-	-
Thr243	290 ± 6	1.28	Tyr150	0.35	-
Ser287	295 ± 15	0.77	Tyr150	0.36	+
Lys402	737 ± 27	0.37	-	-	+
Ser454	572 ± 17	1.14	Tyr411	0.71	+
Thr540	759 ± 21	0.87	Lys402	0.37	+

*K*_d_—the dissociation constant of an obtained complex;dist (P-O(N))—the distance between the phosphorus atom of soman and the hydroxyl oxygen atom (nitrogen in the case of lysine) of an amino acid studied.

**Table 2:** The result of the molecular docking of the isomers of soman into the sites Ser193 and Lys402 of albumin.

Binding Site	Binding Parameters	Stereoisomers of Soman			
		P(−)C(−)	P(+)C(−)	P(−)C(+)	P(+)C(+)
Ser193	*K*_d_, μM	518 ± 14	598 ± 5	787 ± 27	633 ± 7
	dist(P-O(N)), nm	0.38	0.41	0.37	0.40
Lys402	*K*_d_, μM	737 ± 27	902 ± 45	763 ± 25	1031 ± 70
	dist(P-O(N)), nm	0.37	0.43	0.37	0.55

*K*_d_—the dissociation constant of an obtained complex; dist (P-O(N))—the distance between the phosphorus atom of soman and the hydroxyl oxygen atom (nitrogen in the case of lysine) of an amino acid studied.

**Table 3:** The result of molecular docking of paraoxon into the sites Tyr150 (149) and Tyr411 (410) of HSA and BSA.

Binding Site HSA(BSA)	Binding Parameters	HSA	BSA
Tyr150 (149)	*K*_d_, μM	4 ± 0.3	9 ± 0.7
	dist(P-O), nm	0.36	0.37
Tyr411 (410)	*K*_d_, μM	87 ± 5	13 ± 1
	dist(P-O), nm	0.37	0.35

*K*_d_—the dissociation constant of an obtained complex. dist(P-O)—the distance between the phosphorus atom of paraoxon and the hydroxyl oxygen atom of a tyrosine.
